# Prognostic relevance of lactate dehydrogenase in advanced pancreatic ductal adenocarcinoma patients

**DOI:** 10.1186/s12885-016-3012-8

**Published:** 2017-01-06

**Authors:** Yuanyuan Xiao, Wen Chen, Zhihui Xie, Zhenyi Shao, Hua Xie, Guoyou Qin, Naiqing Zhao

**Affiliations:** 1 Department of Biostatistics, School of Public Health, Fudan University, 130 Dong’an Road, Shanghai, China; 2 School of Public Health, Kunming Medical University, Kunming, Yunnan China; 3 Information Center, Shanghai Municipal Commission of Health and Family Planning, Shanghai, China; 4 Key Lab of Health Technology Assessment, Ministry of Health (Fudan University), Shanghai, China

**Keywords:** Lactate dehydrogenase, Advanced pancreatic ductal adenocarcinoma, Survival analysis, Restricted cubic spline

## Abstract

**Background:**

The prognostic role of pretreatment serum lactate dehydronegase (LDH) has been well established in many malignant tumors, albeit it remains under-discussed in pancreatic cancer. In the present study, we aimed to assess the association between baseline LDH levels and overall survival (OS) in advanced pancreatic ductal adenocarcinoma (PDAC) patients who did and did not receive subsequent chemotherapy.

**Methods:**

In total, 135 retrospectively determined patients with locally advanced or metastatic PDAC, who were diagnosed between 2012 and 2013, were analyzed. Baseline LDH levels were detected within 20 days after histopathological confirmation of the diagnosis. Multivariate Cox proportional hazards regression model was applied to estimate the adjusted hazards ratio (HR) for LDH levels and OS of PDAC. We used restricted cubic spline (RCS) to further investigate dose-effect relationship in the association.

**Results:**

Having adjusted for possible confounders, we found that in advanced PDAC patients who went through subsequent chemotherapy, an elevated pretreatment LDH level (≥250 U/L) had an adjusted HR of 2.47 (95% CI = 1.28–4.77) for death, but patients, who did not receive chemotherapy, had no significant HR (adjusted HR = 1.57; 95% CI = 0.83–2.96). RCS fitting results revealed a steep increase in HR for PDAC patients received chemotherapy with a baseline LDH > 500 U/L.

**Conclusions:**

Pretreatment LDH levels had noticeable prognostic value in PDAC patients who received subsequent chemotherapy. Tackling elevated LDH levels before the initiation of chemotherapy might be a promising measure for improving OS of patients after treatment for their advanced PDAC. Studies with a large sample size and a prospective design are warranted to substantiate our findings.

## Background

In metabolic perspective, the most distinctive feature of cancer cells is the enhanced glycolytic activity even under sufficient oxygen supply, which is well known as the “Warburg effect” [1]. As a solid tumor which featured in hypoxia, some newly uncovered evidence has suggested that the “Warburg effect” may play a central role in the initiation, progression, and invasion of pancreatic cancer [2].

In the end of glycolysis process, lactate dehydrogenase (LDH) is involved as the catalyst in transforming pyruvate into lactate. It has been found that *LDHA* gene expression is up-regulated in many human malignant tumors, such as cancers of the esophagus [3], stomach [4], lung [5], colorectum [6], and more recently, pancreas [7].

The over-expression of *LDHA* inevitably promotes the production of LDH by cancer cells. Thus, the prognostic value of serum LDH levels in cancer has long been a topic of considerable research interest. Currently, the hazardous role of an elevated pretreatment LDH levels in survival of patients with small-cell lung cancer, nasopharyngeal cancer, colon cancer, and aggressive lymphoid cancers has been well established [8–13]. However, the association between serum LDH levels and pancreatic cancer survival has only been discussed at a very limited scale, although several published studies reached a consensus in supporting an inverse association [14–19]. Because published studies generally focused on advanced pancreatic cancer patients who received palliative chemotherapy, it is not clear whether the prognostic relevance of baseline LDH levels also exists in patients who are precluded from chemotherapy, which is another issue of potential clinical relevance, albeit it has never been discussed.

In the present study, we aimed to assess the association between baseline LDH levels and overall survival (OS) in advanced pancreatic ductal adenocarcinoma (PDAC) patients who did and did not receive chemotherapy. Moreover, we further analyzed the dose-effect relationship in the association between LDH and OS of PDAC.

## Methods

### Study design

The study population consisted of 135 PDAC patients diagnosed between January 1, 2012 and December 31, 2013. All patients were retrospectively determined in a mega population-based electronic inpatients database originated from Shanghai metropolitan area, China. Other than histopathological confirmation, inclusion criteria for PDAC patients were: 1) locally advanced or metastasis occurred, already missed the opportunity for curative operation; 2) survival length, defined as time interval between the date of diagnosis and the date of death, surpassed 30 days; 3) vital information for analysis, such as age, sex, baseline (defined as within 20 days after PDAC confirmation) serum LDH and albumin test results, and chemotherapy regimens, was complete.

The outcome of interest was OS, and the date of death for PDAC patients was acquired through external matching with death registration system. The deadline of matching was set as January 31, 2015. The study protocol was reviewed and approved by Institutional Research Ethics Board of Fudan University, because of the retrospective nature, plus no individually identifiable or sensitive information was involved, informed consents from all patients had been waived.

General characteristics of 135 PDAC patients we studied are described in Table 1. The mean age of patients was 65.56 years, with a standard deviation of 10.91 years. The numbers of males and females were comparable. The longest survival length of PDAC patients was 965 days, and the shortest was 31 days. The median survival time was 214 days. Means of baseline LDH and albumin levels were 216.04 units/liter (U/L) and 38.61 g/L, respectively. Overall, 68 patients received subsequent chemotherapy, which accounted for 50.37%. Among the 68 patients who received chemotherapy, over 90% received gemcitabine alone or in combination with other agents and over 80% (*N* = 116) died before the pre-designated matching deadline.

**Table 1:** General characteristics of PDAC patients analyzed (*N* = 135)

Characteristic	Mean (Std.)/Median	Count (%)
Age (yrs)	65.56 (10.91)	-
Survival length (days)	214	-
Baseline LDH (U/L)	216.04 (90.96)	-
Baseline albumin (g/L)	38.61 (5.09)	-
Sex (Male)	-	65 (48.15)
Subsequent chemotherapy (Yes)	-	68 (50.37)
Gemcitabine only	-	12 (17.65)
Gemcitabine combined chemotherapy	-	50 (73.53)
Other chemotherapy regimen	-	6 (8.82)

### Variables and definitions

A normal level of serum LDH is usually defined as less than 250 U/L. We used this cutoff-value to dichotomize PDAC patients into “normal LDH” and “elevated LDH” groups based on the baseline test results. Baseline serum albumin levels were defined as “normal” (≥35 g/L) and “decreased” (<35 g/L) accordingly. Palliative chemotherapy was defined as the administration of one or more following medications that are commonly used for treatment of PDAC in China: gemcitabine, nab-Paclitaxel, 5-fluorouracil, Irinotecan, and Oxaliplatin.

### Statistical analysis

Descriptive statistics were used to illustrate or compare characteristics within or between PDAC patients from different baseline LDH groups. Multivariate Cox proportional hazards regression model was applied to estimate the adjusted hazards ratio associated with LDH levels. Finally, considering the arbitrariness which might have been introduced by pre-designated cutoff for baseline LDH levels, a continuous variable, as well as the possibility of nonlinear relationship, we adopted restrictive cubic spline (RCS) to discuss the dose-effect relationship in the association between LDH and death hazards in PDAC patients who did and did not receive chemotherapy separately. We chose three knots to fit RCS: the 5^th^, 50^th^, and 95^th^ percentiles of LDH levels.

All statistical analyses were executed by SAS (version 9.2, SAS Institute Inc., Cary, NC, USA), and the significance level for test or inference was set as two-tailed probability <0.05.

## Results

### Overall survival of PDAC patients with different baseline LDH levels

By applying the aforementioned cutoffs for LDH and albumin levels, 30 (22.22%) patients recorded an elevated baseline LDH level, while 26 (19.26%) presented decreased baseline albumin level. There are significant distributional differences in age at diagnosis and albumin levels between PDAC patients with normal and elevated baseline LDH levels, and the OS was comparatively inferior for PDAC patients with elevated LDH levels (Table 2).

**Table 2:** Distributional differences in general characteristics for PDAC patients of different baseline LDH levels

Characteristics	Normal baseline LDH (*N* = 105)	Elevated baseline LDH (*N* = 30)	*p* value
Age at diagnosis (Yrs, mean, std.)	65.04 (9.94)	67.37 (13.81)	0.02^a^
Sex (Male, %)	51 (48.57)	14 (46.67)	1.00^b^
Baseline albumin (Deceased, %)	16 (15.24)	10 (33.33)	0.04^b^
Subsequent chemotherapy (Yes, %)	55 (52.38)	13 (43.33)	0.41^b^
Survival length (Days, Median)	238	96	<0.01^c^

^a^By *t* test ^b^By Fisher’s exact test ^c^By log-rank test

**Fig. 1:**
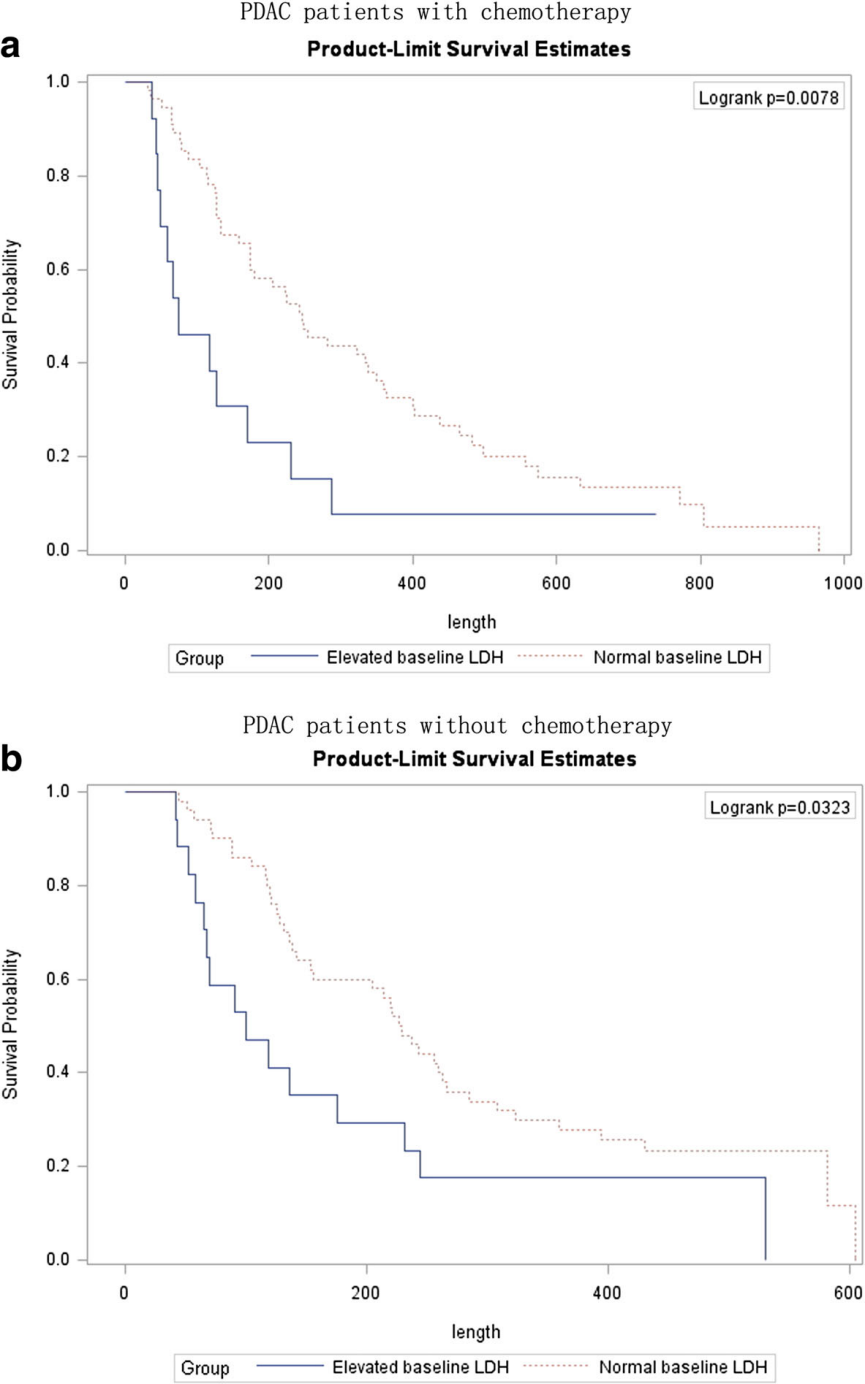
Kaplan-Meier curves illustrating overall survival status by baseline LDH levels in two groups of advanced PDAC patients. **a** PDAC patients with chemotherapy; **b** PDAC patients withoutchemotherapy

For subsequent chemotherapy, we sketched Kaplan-Meier survival curves with regard to baseline LDH levels: for both groups of patients, an elevated baseline LDH level was associated with significantly compromised OS (Fig. 1).

### Independent association between baseline LDH levels and OS of PDAC

The serum LDH level is also an indicator of liver function, and because of anatomic vicinity, advanced pancreatic cancer usually metastases to the liver, leading to a decreased liver function in turn [20]. Therefore, when constructing multivariate Cox model, we further included the serum albumin level, a more sensitive indicator of liver function to adjust for possible confounding. Cox model fitting results are enumerated in Table 3. For advanced PDAC patients who underwent subsequent palliative chemotherapy, univariate Cox model found that pretreatment LDH level was significantly associated with deteriorated survival (crude HR = 2.34; 95% CI = 1.23–4.45), and this notable association was maintained in the multivariate model: compared with patients having a normally ranged LDH level, an elevated LDH level was associated with an HR of 2.47 (95% CI = 1.28–4.77). For PDAC patients who did not accept chemotherapy, initially a prominent association between baseline LDH levels and OS has been discerned (crude HR = 1.91; 95% CI = 1.04–3.47), but after adjustment for possible confounders, this association was insignificant (adjusted HR = 1.57; 95% CI = 0.83–2.96).

**Table 3:** Multivariate Cox regression model fitting results by acceptance of chemotherapy in advanced PDAC patients

Independent variables	Chemotherapy group		Non-chemotherapy group	
	Crude HR (95% CI)	Adjusted HR (95% CI)	Crude HR (95% CI)	Adjusted HR (95% CI)
Age at diagnosis (+5 years)	1.09 (0.94–1.25)	1.06 (0.94–1.20)	1.17 (1.03–1.33)	1.14 (1.00–1.30)
Sex (Male)	0.64 (0.38–1.07)	0.67 (0.39–1.14)	0.90 (0.53–1.55)	0.89 (0.50–1.56)
Baseline albumin (Decreased)	3.14 (1.67–5.89)	3.27 (1.70–6.27)	1.24 (0.62–2.47)	1.06 (0.51–2.21)
Baseline LDH (Elevated)	2.34 (1.23–4.45)	2.47 (1.28–4.77)	1.91 (1.04–3.47)	1.57 (0.83–2.96)

### Dose-response association between baseline LDH and OS of PDAC

**Fig. 2:**
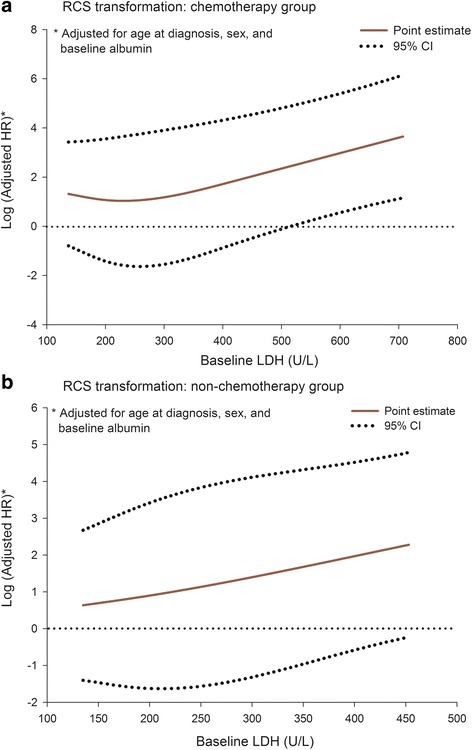
RCS fitting results for baseline LDH and OS in two groups of advanced PDAC patients. **a** chemotherapy group; **b** non-chemotherapy group

RCS fitting results disclosed that, in general, the dose-response trend between pretreatment LDH levels and HR was less apparent. In advanced PDAC patients who received chemotherapy, LDH levels higher than 500 U/L were associated with a significantly increased HR. On the contrary, in PDAC patients who did not receive chemotherapy, the change of baseline LDH levels showed an insignificant influence on OS (Fig. 2).

## Discussion

In the present study, we discussed the influence of LDH levels measured right after cancer diagnosis on OS of patients with advanced PDAC. Based on multivariate Cox model and RCS, we observed that, for patients who received subsequent palliative chemotherapy, an elevated baseline LDH level was associated with nearly 2.5 folds hazards of death, whereas for patients who did not receive chemotherapy, this association was not statistically significant.

Serum LDH levels are widely accepted as indicators of tissue breakdown. In cancer patients, because of enhanced proliferation capacity, the cycle of cancer cells will be shortened, which in turn causes an increased risk of necrosis. Besides, vicinal normal tissues can be encroached upon and destructed by cancer cells [21]. All these mechanisms, along with enhanced glycolysis, may collectively contribute to increased serum LDH levels in cancer patients. In this sense, LDH levels can actually partly reflect tumor burden. It has been suggested that, high concentration of lactate can promote tumor progression and metastasis through up-regulation of tumor growth factors, such as vascular endothelial growth factor and hypoxia-inducible factor 1α, or through the direct enhancement of cellular motility [22]. More recently, Rong et al*.* found that LDH directly promotes the growth of pancreatic cancer cells [7]. Thus, it is reasonable to suspect that the significant inverse association between baseline LDH levels and survival we found in advanced PDAC patients who received chemotherapy can partly be attributed to tumor burden or pro-progression nature of LDH. As to the reason that why this association was not recognizable in patients who did not receive chemotherapy, the most likely explanation is that, usually an end-stage disease and exhausted physical status are major hurdles that may prevent cancer patients from chemotherapy; therefore, in this group of patients, the plummeting health would inundate a comparatively weak influence of LDH in survival, if it indeed existed.

High serum LDH levels have been found to be associated with resistance to chemotherapy in many types of cancer, such as cancers of the colorectum [23, 24], breasts [25], and lung [26, 27], just to name a few. A popular theory for this phenomenon is that stromal cells inversely transform lactate into pyruvate, which fuels progression of cancer cells and strengthens their resistance to chemotherapeutic agents [1, 28]. One previously published in vitro study clearly demonstrated that, novel LDH inhibitors exhibited synergistic cytotoxic activity with gemcitabine [29]. Therefore, it might be true that the association between elevated pretreatment LDH levels and deteriorated survival in advanced PDAC patients who went through chemotherapy was actually the association between enhanced chemoresistance and increased hazards of death.

Nevertheless, either way suggests the promising role of baseline LDH levels in individualized treatment of PDAC: for patients who are designated for chemotherapy, tackling elevated LDH levels before treatment may alleviate tumor stress and improve the efficacy of chemotherapeutic agents, thus gain survival benefit in the end. Currently, various effective LDH inhibitors are already available, and the inhibition of LDH has minimum impact on normal tissues and presents no major side effects [29–31]. More importantly, the reduction in LDH activity has been proved an effective anti-proliferation measure for several other types of cancer in vivo [32, 33]. For PDAC patients who are not suitable for chemotherapy, based on current evidence, the therapeutic value of LDH inhibition cannot be concluded yet.

Although the acceptance of chemotherapy can be an ideal surrogate for the disease stage and physical performance status, there lies a possibility that cost concern prevented some eligible PDAC patients from this available but expensive treatment, and this situation could introduce bias to the association between baseline LDH levels and OS in patients who did not receive chemotherapy. Nonetheless, this bias tended to derail the association away from the null, and even so, we still concluded an insignificant association in this group of patients.

Several limitations of the present study should be considered. At first, the risk of selection bias cannot be eliminated as we only chose advanced PDAC patients whose vital information was complete. Besides, although when estimating the association between baseline LDH levels and PDAC survival, we have successfully controlled for multiple possible confounders, residual confounding effect undoubtedly existed, and its extent is hard to estimate. Finally, all patients were originated from a localized region in China, thus the generalization of our study results should be made with caution. For future studies, from the genetic perspective, the association between LDH gene expression and OS of both advanced and resectable PDAC patients is a promising topic that deserves additional investigation. It is critically important to unveil the possible underlying mechanisms of our findings and to successfully implement effective intervention measures in improving the prognosis of PDAC patients.

## Conclusions

In this retrospective study, we assessed the association between LDH levels measured right after cancer diagnosis and OS in a group of advanced PDAC patients. We found that an elevated pretreatment LDH level was associated with significantly deteriorated survival in PDAC patients who received subsequent chemotherapy, but this association was not statistically significant in PDAC patients who did not receive chemotherapy. Our findings suggest that, for PDAC patients, before the initiation of chemotherapy, tackling the enhanced LDH activity may ultimately improve survival. Prospective cohorts are warranted to validate our findings.

## Abbreviations

HR: Hazard ratio
LDH: Lactate dehydrogenase
OS: Overall survival
PDAC: Pancreatic ductal adenocarcinoma
RCS: Restricted cubic spline
